# Improving allergy management and treatment: a proposed algorithm and curriculum for prescribing allergen immunotherapy in the primary care setting

**DOI:** 10.1038/s41533-024-00380-z

**Published:** 2024-08-01

**Authors:** Giseth Bustos, Marcos A. Sanchez-Gonzalez, Troy Grogan, Adriana Bonansea-Frances, Camysha Wright, Frank Lichtenberger, Syed A. A. Rizvi, Alan Kaplan

**Affiliations:** 1 Universidad El Bosque, Medicine, Bogota, Colombia; 2 Lake Erie College of Osteopathic Medicine, Bradenton, FL USA; 3 MedScience Research Group, Inc., West Palm Beach, FL USA; 4 Florida Center For Allergy & Asthma Care, Homestead, FL USA; 5 ENT & Allergy Associates of Florida, Plantation, FL USA; 6 Piedmont Healthcare, Statesville, NC USA; 7 College of Biomedical Sciences, Larkin University, Miami, FL USA; 8 Family Physician Airways Group of Canada, Ontario, Canada

**Keywords:** Immunotherapy, Asthma

## Abstract

Allergic rhinitis (AR), a condition characterized by sensitivity to allergens leading to poor quality of life, including disrupted sleep, reduced vitality, lowered mood, changes in blood pressure limited frustration tolerance, impaired focus, decreased performance in academic and professional settings, and millions of missed work and school days every year. Approximately 20–40% of individuals in the United States are affected by AR, which carries notable clinical and financial burdens. Interestingly, there is a strong link between AR and asthma to the extent that countries with a high prevalence of rhinitis have asthma rates ranging from 10% to 25%. Research has indicated that Allergen Immunotherapy (AIT) is associated with improved AR symptoms, a potential to resolve the AR over time, a decreased likelihood of asthma exacerbations and incidence of pneumonia in individuals with concurrent asthma, which are advantages that persist for years even after the cessation of treatment. Although patients presenting with allergies are first seen and treated in the primary care setting, gaps in training and the lack of available guidance for primary care practitioners have significantly impacted the quality of care for these patients with persistent AR symptoms, resulting in inefficient use of healthcare resources. To complicate matters, there is an insufficiency of allergists and immunologists, impacting the capacity to provide next-level care to the number of AR patients who could benefit from AIT. Hence, there is a critical need to equip primary care providers with educational experiences on essential concepts related to immune responses in allergies and asthma, recognizing the significance of the common airway in treating these entities and familiarization with the scientific evidence supporting various options for AIT. The development and implementation of medical education and algorithms designed to assess diverse patients’ symptoms, pharmacotherapy approaches, and situations where AIT can be initiated or sustained are warranted. The present commentary proposes a workflow model of the critical steps for managing and treating mild to moderate respiratory allergies via AIT in primary care settings. In addition, the initial development of medical education programs to minimize the burden on allergy-specialized care while, importantly, actively improving patient outcomes will be discussed.

## Introduction

Allergic rhinitis (AR) is a prevalent condition characterized by increased sensitivity to otherwise benign allergens, resulting in a range of adverse effects on an individual’s quality of life. Some more persistent AR effects include disrupted sleep, reduced vitality, lowered mood, limited frustration tolerance, impaired focus, and decreased academic and professional performance, significantly stressing healthcare systems’ clinical and financial aspects^1^. In the United States, approximately 20–40% of the population is affected by AR, further underscoring the significance of this issue^2^.

The link between AR and asthma, another inflammatory airway condition, is becoming increasingly evident owing to the comorbid nature of these entities, ranging from 10% to 25%^3^. Although our understanding of the link between AR and asthma has advanced over the last few years, there is consensus to suggest that the upper and lower airways are a unified morphological and functional unit in health and disease^3^. In support of the abovementioned notion, the Allergic Rhinitis and Its Impact on Asthma (ARIA) guidelines, initially published in 2001, highlight the importance of appropriate management of AR in patients with asthma^4,5^. Moreover, recent research has shed light on the potential benefits of Allergen Immunotherapy (AIT) in alleviating AR symptoms, possibly leading to long-term resolution of the condition and a reduction in asthma exacerbations and incidence of pneumonia^6^. Interestingly, AIT appears to confer advantages that persist even after treatment cessation. In this vein, analyzing cost-effectiveness data from a Florida Medicaid population, it is apparent that the total average healthcare cost over 18 months can be reduced by 38% for patients receiving AIT compared to those receiving standard care or $6637 versus $10,644 USD, respectively with reductions that were observed in both adult (30%) and pediatric (42%) groups^7^.

The current primary healthcare landscape presents challenges in providing optimal care to AR patients. To complicate matters, there is a shortage of allergists and immunologists, limiting the availability of specialized care for the increasing number of patients who could benefit from AIT^8^. Primary care practitioners often encounter and treat patients with allergies initially. However, gaps in their training and a lack of clear guidance have hindered the effective management of persistent symptoms, resulting in inefficient resource utilization. Accordingly, there is a critical need to equip primary care providers with the corresponding training. This commentary proposes a lucid, comprehensive workflow model for primary care practitioners to address the challenges of treating mild to moderate respiratory allergies and using AIT to manage patients when appropriate. The model aims to guide practitioners through critical steps in patient assessment, pharmacotherapy approaches, and initiating or continuing AIT. Additionally, the paper emphasizes the importance of developing medical education programs and bridging the gap between primary care and allergy-related treatment, which seeks to provide more effective and efficient care for AR patients.

## Proposed model

One of the main goals of establishing a primary care-based treatment workflow algorithm is to identify, treat, and initiate AIT. The present work aligns with identified needs and, per established clinical guidelines, systematically formulated an algorithm for patients presenting with symptoms suggestive of respiratory allergic diseases commonly encountered in primary care settings. It is important to note that patients considered for AIT have been initially treated for allergic rhinitis but have not responded to the treatment. The algorithm was developed to bridge the knowledge gap created by the high prevalence of allergies with high patient demand, insufficient allergy specialists, and the primary care physicians’ lack of knowledge and confidence to tackle this salient healthcare problem.

Initially, the algorithm highlights the interpretation of the symptoms found in patients with allergic rhinitis with or without allergic conjunctivitis and/or asthma, depending on the case. The abovementioned interpretation will be based on the visual analog scale (VAS) and other instruments, including those previously described, such as the daily symptoms score (DSS), the sino-nasal outcome test (SNOT-22), total nasal symptom score (TNSS), the rhinitis control assessment test (RCAT)^1,9–13^. The second step in the algorithm is focused on treatment, which may include oral, intranasal, and/or ocular antihistamines, intranasal steroids, leukotriene inhibitors, and cromolyn. The use and combination of medications are at the clinical discretion of the clinician, depending on the particular case. The last step in the patient’s symptomatic evaluation is identifying whether their allergy is perennial or seasonal. Having determined the previous relationship, confirmatory allergy tests will be performed to determine the following intervention path.

**Fig. 1:**
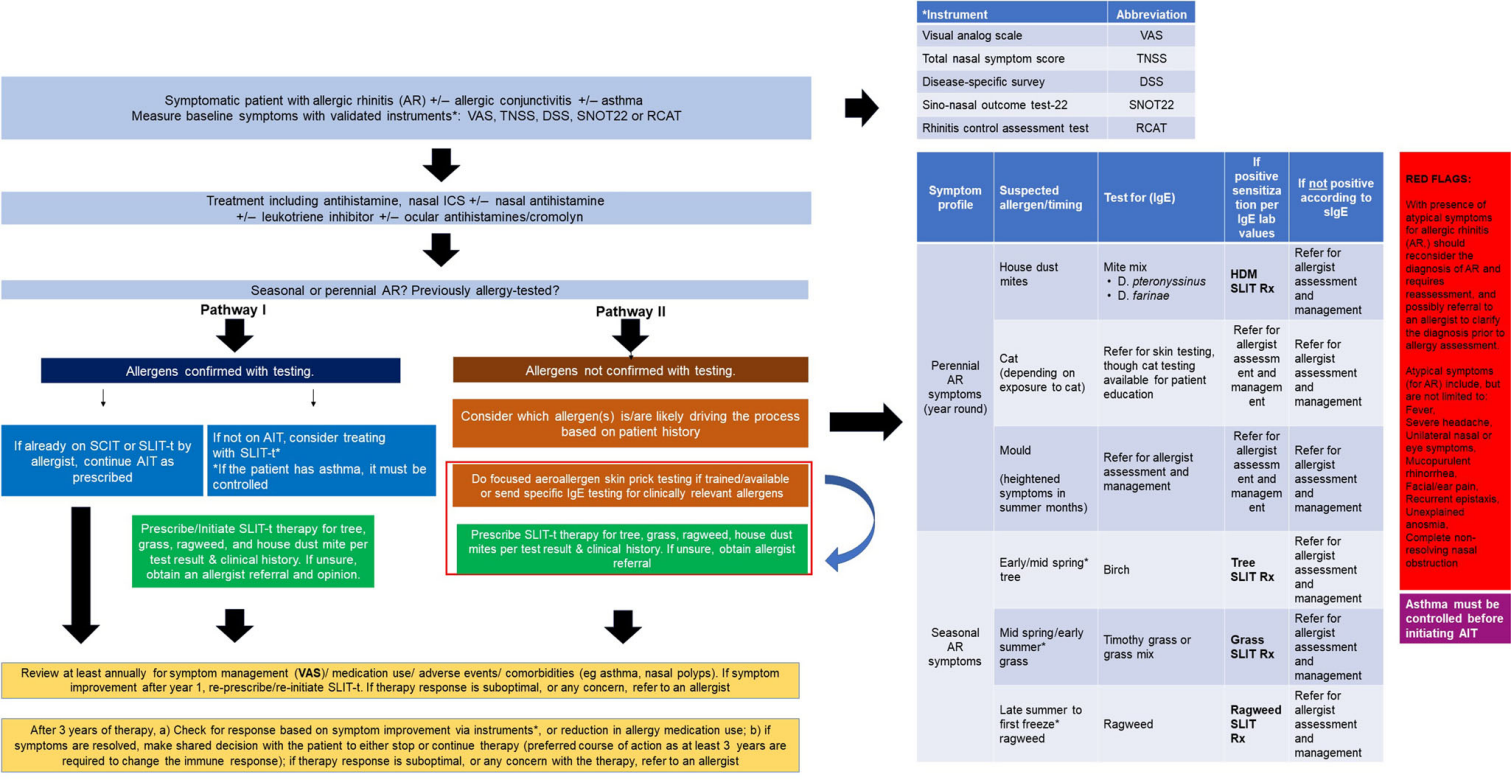
Proposed algorithm for prescribing allergen immunotherapy in the primary care setting.
- *Pathway II*—When allergy testing has not yet been done to confirm allergen positivity, the PCP must consider which allergens are probably more relevant according to the patient’s clinical history and region. Then, the appropriate testing should be performed. testing could include skin tests if the physician is trained and/or measurement of serum IgE to the specific allergen. Subsequently, appropriate Sublingual AIT for tree, grass, ragweed, and house dust mites should be considered for prescription. It is worth noting that compared to subcutaneous immunotherapy (SCIT), sublingual AIT (SLIT) is considered safer with a much lower risk of anaphylaxis (James & Bernstein)^15^.
- *Long-term AIT efficacy*—After three years of therapy, the PCP should check for response based on symptom improvement via validated instruments of choice (e.g., VAS, TNSS, SNOT22, RCAT) or reduce allergy medication use. If symptoms are resolved, make a shared decision with the patient to stop or continue therapy (preferred course of action as at least three years are required to change the immune response). If the therapy response is suboptimal, or if there is any concern with the therapy, the primary refer to an allergist care practitioner should refer to an allergist when in doubt.

- *Patient selection*—The present AIT algorithm is not indicated for uncontrolled asthma or anaphylaxis risk if it cannot be managed mainly for specific allergens that are common and easily diagnosed and managed per established guidelines for AIT^14^.
- *Pathway I*—When the test confirms the allergy trigger, the first path will be to investigate whether the patient is already under subcutaneous or sublingual AIT by the allergist, after which it will be decided to continue the management. If the patient is not on allergy immunotherapy, it is considered to start AIT. If the patient is diagnosed with asthma, it must be controlled before initiating immunotherapy (Fig. 1).

## Medical education needs

An objective description of the deficits of the intended learners’ current knowledge or practice gaps and a description of the ideal knowledge practice in the management and application of AIT is warranted. The authors of the present commentary have identified the need to encourage primary care physicians and pediatricians to improve allergy diagnosis, management, and AIT-related skills. The need for more value-based models has been well-documented in the literature over the past two decades^16–18^. Unfortunately, our preliminary gap analyses demonstrate that primary care practitioners often indicate limited training in aspects revolving around allergy management.

## Curriculum development

The algorithm has been considered an initial step in developing educational strategies to improve basic allergic AIT skills and discuss tactics to diagnose, treat, and manage AR in the primary care setting. Hence, the medical education curriculum was developed to emphasize acquiring clinical skills to provide quality care for those suffering from allergies, among the most critical and prevalent medical entities encountered by primary care practitioners.

**Fig. 2:**
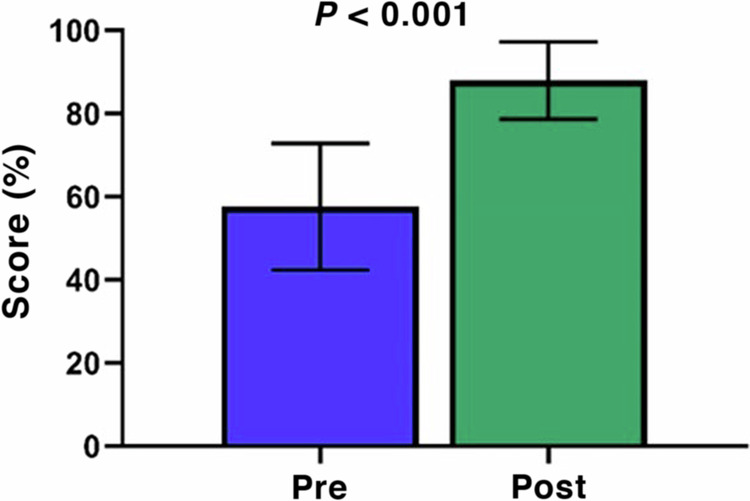
Pretest post-test paradigm demonstrating improved participants’ allergy immunotherapy knowledge in response to the educational activity.

- *Educational gap*—The primary care practitioners’ crucial role in managing allergic rhinitis and asthma has been recognized for years. The present curriculum aims to bridge the gap created by the shortage of board-certified allergists. Given that allergic rhinitis affects a significant portion of the U.S. population, leading to considerable clinical and economic burdens, the upskilling of non-specialists is imperative for adequately managing these common conditions.
- *Importance of the topic and rationale*—The curriculum prioritizes AIT due to its established efficacy and safety profile, addressing the need for quality allergy care beyond the specialist setting. Unfortunately, a shortage of board-certified allergists has been reported to address the number of patients suffering from A.R. who could benefit from AIT^8^. By equipping non-specialists with this knowledge, the program endeavors to decentralize quality allergy care and make it more accessible to patients suffering from respiratory allergies.
- *Educational goals and objectives*—Using Kern’s six-step model, a curriculum was meticulously designed to enhance healthcare providers’ understanding of and management skills in allergies and AIT^19^. In addition, the curriculum covers immune response mechanisms, cutting-edge treatment methods, and strategies for effective patient counseling, aiming to foster a comprehensive approach to allergy management.
- *Educational strategy* *and* *instructional design*—A variety of educational techniques were employed, including interactive lectures of 30 to 90 min each (6 h total), case study discussions, ensuring participants gain a practical understanding of AIT and its application in clinical practice. This multimodal approach caters to different learning styles and reinforces the knowledge to manage allergies effectively.
- *Course evaluation and assessments*—The curriculum’s effectiveness was assessed utilizing a pre-and post-test paradigm with 10 multiple-choice items, revealing approximately 20% (Fig. 2) improvement in participants’ knowledge. This metric measures educational outcomes and reflects the program’s ability to address the identified gaps. The curriculum seems to significantly bolster the competencies of participants in allergy management through the use of AIT, reflecting the program’s success as demonstrated by the pre-post test assessments (*P* < 0.001).

## Conclusions

The proposed algorithm was designed to assist primary care providers in managing allergic rhinitis with or without current allergic conjunctivitis and asthma to prescribe allergen immunotherapy correctly and safely. The tools were thought to be easily accessible and intuitive while emphasizing the importance of developing medical education programs to bridge the gap between current primary care with limited options and the necessary treatments that can be addressed by continuing medical education. The purpose is to build the confidence of the physicians prescribing effective disease-modifying allergen immunotherapy and provide timely access to patients requiring it. In addition to using existing tools and providing the physician with sufficient knowledge, we can build this expertise with allergy-focused patient histories, choose the most appropriate laboratory and confirmatory tests, and initiate/continue sublingual immunotherapy, depending on the case. Achieving easy and rapid access to health services will improve resource use in a cost-effective framework with important implications for quality of care and healthcare costs. Developing medical education programs such as the one in the present article to equip primary care practitioners with the tools and essential knowledge to treat and manage AR is warranted. Although it is early to determine the main impact of the above algorithm and educational activity, anecdotal experience suggests that many educational activity participants are successfully adopting these practices in their clinics. Future research will also evaluate the long-term effects on patient care and determine the curriculum’s and proposed algorithm’s impact on the quality of life for those with respiratory allergies.
